# Eco-epidemiology of *Borrelia miyamotoi* and Lyme borreliosis spirochetes in a popular hunting and recreational forest area in Hungary

**DOI:** 10.1186/s13071-015-0922-2

**Published:** 2015-06-06

**Authors:** Sándor Szekeres, Elena Claudia Coipan, Krisztina Rigó, Gábor Majoros, Setareh Jahfari, Hein Sprong, Gábor Földvári

**Affiliations:** Department of Parasitology and Zoology, Faculty of Veterinary Science, Szent István University, 2 István Street H-1078, Budapest, Hungary; Laboratory for Zoonoses and Environmental Microbiology, National Institute for Public Health and Environment, 9 Antonie van Leeuwenhoeklaan, P.O. Box 1, Bilthoven, The Netherlands

**Keywords:** *Borrelia miyamotoi*, *Borrelia burgdorferi* sensu lato, Ticks, *Ixodes acuminatus*, Rodents, *Apodemus flavicollis*, Endophilic pathogen cycle, Hunters, Hungary

## Abstract

**Background:**

*Borrelia miyamotoi,* the newly discovered human pathogenic relapsing fever spirochete, and *Borrelia burgdorferi* sensu lato are maintained in natural rodent populations. The aim of this study was to investigate the natural cycle of *B. miyamotoi* and *B. burgdorferi* s.l. in a forest habitat with intensive hunting, forestry work and recreational activity in Southern Hungary.

**Methods:**

We collected rodents with modified Sherman-traps during 2010–2013 and questing ticks with flagging in 2012. Small mammals were euthanized, tissue samples were collected and all ectoparasites were removed and stored. Samples were screened for pathogens with multiplex quantitative real-time polymerase chain reaction (qPCR) targeting a part of flagellin gene, then analysed with conventional PCRs and sequencing.

**Results:**

177 spleen and 348 skin samples of six rodent species were individually analysed. Prevalence in rodent tissue samples was 0.2 % (skin) and 0.5 % (spleen) for *B. miyamotoi* and 6.6 % (skin) and 2.2 % (spleen) for *B. burgdorferi* s.l. Relapsing fever spirochetes were detected in *Apodemus flavicollis* males, *B. burgdorferi* s.l. in *Apodemus* spp. and *Myodes glareolus* samples. *Borrelia miyamotoi* was detected in one questing *Ixodes ricinus* nymph and *B. burgdorferi* s.l in nymphs and adults. In the ticks removed from rodents DNA amplification of both pathogens was successful from *I. ricinus* larvae (*B. miyamotoi* 5.6 %, *B. burgdorferi* s.l. 11.1 %) and one out of five nymphs while from *Ixodes acuminatus* larvae, and nymph only *B. burgdorferi* s.l. DNA was amplified. Sequencing revealed *B. lusitaniae* in a questing *I. ricinus* nymph and altogether 17 *B. afzelii* were identified in other samples. Two *Dermacentor marginatus* engorged larva pools originating from uninfected hosts were also infected with *B. afzelii*.

**Conclusions:**

This is the first report of *B. miyamotoi* occurrence in a natural population of *A. flavicollis* as well as in Hungary. We provide new data about circulation of *B. burgdorferi* s.l. in rodent and tick communities including the role of *I. acuminatus* ticks in the endophilic pathogen cycle. Our results highlight the possible risk of infection with relapsing fever and Lyme borreliosis spirochetes in forest habitats especially in the high-risk groups of hunters, forestry workers and hikers.

## Background

Rodents support tick populations by providing a stable blood source as well as serve as important reservoirs for tick-borne pathogens [1, 2]. Exophilic (or non-nidicolous) ticks, such as all stages of *Ixodes ricinus* and adult *Dermacentor marginatus*, await a host on the vegetation, and are usually less host-specific, thus may act as bridge vectors between small mammals and humans in natural or urban habitats [3–5]. Therefore, understanding the epidemiology of these zoonotic agents in rodents and ticks has great public health relevance.

People working in the forests (e.g. hunters and forestry workers) and hikers have the highest probability to get into contact with ticks and therefore, with tick-borne pathogens. The seroprevalence of tick-borne pathogens in forestry workers has been reported to be higher compared to the average population in Europe, including Hungary [6, 7]. We have relatively few data on the infection rates of hunters with tick-borne pathogens and consequent risk of tick-borne diseases [8–13].

Human pathogenic members of the genus *Borrelia* consist of two main groups of spirochetes. The first, the causative agents of Lyme borreliosis (LB), is widespread throughout the Northern Hemisphere and transmitted by members of the *Ixodes ricinus* complex, while the second group, causing relapsing fever (RF) in humans, is transmitted by soft ticks, hard ticks [14] and lice [15]. *Borrelia miyamotoi*, belonging to the relapsing fever group, is transmitted by the same *Ixodes* species that also transmit LB spirochetes and is the only known agent causing relapsing fever transmitted by hard ticks*. Borrelia miyamotoi* was isolated for the first time in Japan in 1995 from *Ixodes persulcatus* ticks as well as from *Apodemus argenteus* mice [16, 17] and, over the last decade, it has also been detected in *I. ricinus* ticks throughout Europe [18–22]. Its ability to cause disease was unknown [23] until the first human cases of *B. miyamotoi* infection were reported in Russia in 2011 [24] and, more recently, in the USA and the Netherlands [25, 26].

Pathogenic members of *B. burgdorferi* s.l. - *B. afzelii, B. garinii, B. burgdorferi* s.s., *B. bavariensis* and *B. spielmanii* - are the causative agents of Lyme borreliosis, which is the most prevalent vector-borne disease in the temperate zone of the Northern Hemisphere. A further three species of the *B. burgdorferi* s.l. complex (*B. bissettii, B. lusitaniae* and *B. valaisiana*) have only occasionally been detected in patients [27]. These bacteria can cause various serious dermatological, rheumatological and neurological symptoms. In Hungary, 947–1811 patients are reported yearly to have LB [28]. Considering other European and North- American data the estimated LB incidence may be ten times higher in Hungary [29].

Based on the high seroprevalence of *B. miyamotoi* in forestry workers, reported in the Netherlands [6], and the relatively common occurrence of the relapsing fever spirochetes in questing ticks in Europe [30, 31], *B. miyamotoi* infection probably also occurs in Hungary. However, the currently used diagnostic methods for patients are not suitable for detecting these spirochetes. A recent seroepidemiological study in the Netherlands showed that forestry workers and patients suspected for human granulocytic anaplasmosis have significantly higher seroprevalence of *B. miyamotoi* compared to the average population [6]. They suggest that some LB patients might also have *B. miyamotoi* infection (either undiagnosed or misdiagnosed or asymptomatic).

We have, also, sporadic information about the natural cycle of *B. miyamotoi. Borrelia miyamotoi* has so far been detected only from *Apodemus argenteus* (small Japanese field mouse) from Japan [17], *Peromyscus leucopus* (white-footed mouse) from USA [32] and *Myodes glareolus* (bank vole) from France [31]. Based on xenodiagnostic experiments of Burri et al. (2014), *Myodes glareolus* and *Apodemus flavicollis* (yellow-necked field mouse) are proven reservoirs of *B. miyamotoi* [33], and *A. argenteus* and *P. leucopus* are candidate reservoir species. Up to date, no other eco-epidemiological studies focusing on the natural cycle of *B. miyamotoi* in Europe were performed.

The aim of this study was to investigate the occurrence of causative agents of relapsing fever and Lyme borreliosis in rodents and ticks in a natural habitat of Southern Hungary, where forestry works, hunting and recreational activities are intensive.

## Methods

Our study site, Gemenc (46° 07′ N, 18° 46′ E), is a floodplain habitat in the southern part of the Hungarian Danube flow. This area has an intensive wildlife management with high density of game animals (red deer, roe deer, fallow deer and wild boar), and is frequently visited by forestry workers, hunters and tourists. According to the data of the Gemenc Forest and Game Co. Ltd., 700 hunters visited the area between September 2014 and February 2015. There are 600 forestry and maintenance workers who enter the forest on a daily basis and approximately 50000 tourists visit the area with 1500 children in summer camps yearly (Károly Árva, personal communication). The details of the sample collection (small mammal trapping and tick flagging) and DNA extraction have been described elsewhere [34]. Engorged larvae from the same tick species removed from the same rodent individual were pooled; all other samples were processed individually.

To determine whether tissue or tick samples contained any pathogens, we used a multiplex quantitative real-time PCR (qPCR) designed for *B. miyamotoi* and *B. burgdorferi* s.l. [35]. Briefly, we targeted a part of the flagellin B (flaB) gene. For *B. miyamotoi* we used forward primer FlabBm.motoiF (5' AGAAGGTGCTCAAGCAG 3') reverse primer FlabB.m.motoiR (5' TCGATCTTTGAAAGTGACATAT 3'), with the probe FlabBm.motoiPro (5' AGCACAACAGGAGGGAGTTCAAGC 3') [26]. For *B. burgdorferi* s.l. we used forward primer B-FlaB-F (5' CAGAIAGAGGTTCTATACAIATTGAIATAGA 3') and reverse primers B-FlaB-Rc (5' GTGCATTTGGTTAIATTGCGC 3') and B-FlaB-Rt (5' GTGCATTTGGTTAIATTGTGC 3'), with the probe B-FlaB-P (5' CAACTIACAGAIGAA XTAAIAGAATTGCTGAICA 3', X = black hole quencher) [35]. In the analysis of qPCR results we selected the positive samples by two criteria, the shape of curves (compared to positive controls) and CT (threshold cycle) values. Samples were considered positive with CT values below 38 cycles for *B. miyamotoi* and below 41 cycles for *B. burgdorferi* s.l.

All qPCR-positive samples were examined by conventional PCR and sequencing. We targeted the glycerophosphodiester phosphodiesterase gene (glpQ) of *B. miyamotoi* with forward primer glpQ-BM-F2 (5' ATGGGTTCAAACAAAAAGTCACC 3') and reverse primer glpQ-BM-R1 (5' CCAGGGTCCAATTCCATCAGAATATTGTGCAAC 3') [26]. We amplified the intergenic spacer region (IGS) of *B. burgdorferi* s.l. with forward primer B5Sborseq (5'-GAGTTCGCGGGAGAGTAGGTTATTGCC-3') and reverse primer B23Sborseq (5'-TCAGGGTACTTAGATGGTTCACTTCC-3') [36]. In the PCR assay we used negative controls to verify and exclude any contaminations. All samples that were positive by conventional PCR have been submitted to sequencing.

For statistical analysis, R [37] and Quantitative Parasitology 3.0 [38] statistical programs were used. Results with p-values under 0.05 were considered significant.

## Results

Tissue samples of six rodent species were individually collected and analysed: *A. flavicollis* (skin: 102, spleen: 67), *A. agrarius* (skin: 202, spleen: 92), *Myodes glareolus* (skin: 29, spleen: 11), *Microtus arvalis* (skin: 7, spleen: 4), *Micromys minutus* (skin: 3), *Mus musculus* (skin: 5, spleen: 3).

Altogether, 8 % of rodents were infested with ticks. The overall prevalence in rodent tissue samples was 0.3 % (skin) and 0.5 % (spleen) for *B. miyamotoi* and 6.6 % (skin) and 2.3 % (spleen) for *B. burgdorferi* s.l*.* (Table 1). *Borrelia miyamotoi* was detected in two *A. flavicollis* males. *Borrelia burgdorferi* s.l. was found in *A. flavicollis*, *Apodemus agrarius* and *My. glareolus* samples.

**Table 1:** Occurrence of *B. miyamotoi* and *B. burgdorferi* s.l. in rodent tissue samples from Southern Hungary^a^

Rodent species	*B. miyamotoi*		*B. burgdorferi s.l.*	
	*(+/tested/prevalence)*			
	skin	spleen	skin	spleen
*A. flavicollis*	1/102/0.9 %	1/67/1.5 %	6/102/5.8 %	3/67/4.5 %
*A. agrarius*	0/202/-	0/92/-	16/202/7.9 %	1/92/1 %
*My. glareolus*	0 /29/-	0 /11/-	1/29/3.5 %	0/11/-
*Mi. arvalis*	0 /7/-	0 /4/-	0 /7/-	0/4/-
*M. minutus*	0 /3/-	-	0 /3/-	-
*Mu. musculus*	0 /5/-	0 /3/-	0 /5/-	0/3/-
Sum	1/348/0.3 %	1/177/0.5 %	23/348/6.6 %	4/177/2.3 %

^a^Skin and spleen samples were not taken from the same individual

In questing *Ixodes ricinus* (21 nymphs and 13 adults)*, B. burgdorferi* s.l was detected in three nymphs and five adults and *B. miyamotoi* was detected in one nymph (Table 2).

**Table 2:** Prevalence of *B. miyamotoi* and *B. burgdorferi* s.l. in questing ticks collected in Southern Hungary

Tick species and stage	*B. miyamotoi*	*B. burgdorferi* s.l.
	*(+/tested/prevalence)*	
*I. ricinus* females	0/5/-	2/5/40 %
*I. ricinus* males	0/8/-	3/8/37.5 %
*I. ricinus* nymphs	1/21/4.8	3/21/14.3 %
*D. reticulatus* ^a^	0/64/-	0/64/-
*D. marginatus* ^a^	0/2/-	0/2/-
*H. concinna* ^b^	0/62/-	0/62/-

^a^adults

^b^33 larvae, 10 nymphs and 19 adults

In the four tick species removed from rodents, *B. miyamotoi* was detected in engorged *I. ricinus* larvae and *B. burgdorferi* s.l. was detected in engorged *I. ricinus* larvae and a nymph*, I. acuminatus* larvae and a nymph, and *D. marginatus* larvae (Table 3). The two *B. miyamotoi*-positive *I. ricinus* larva pools originated from two *A. flavicollis* males with unknown infectious status. Developmental stage and host infectious status for sequenced *B. burgdorferi*-positive *I. ricinus* samples are shown in Table 4. Two *I. acuminatus* larva pools originated from *A. flavicollis* hosts with unknown infectious status and one larva pool and one nymph were removed from uninfected *A. flavicollis* hosts. In the ticks removed from rodents DNA amplification of both pathogens was successful from *I. ricinus* larvae (*B. miyamotoi* 5.6 %, *B. burgdorferi* s.l. 11.1 %) while from 2 *Ixodes acuminatus* larvae (7.7 %), and the single tested nymph only *B. burgdorferi* s.l. DNA was amplified. There was no significant difference in *B. burgdorferi* s.l. minimum infection prevalence between *I. ricinus* and *I. acuminatus* larvae (p > 0.05). Three *Dermacentor marginatus* larva samples (two pools and one single; 4.5 % minimum infection prevalence) removed from two uninfected *A. flavicollis* and an uninfected *A. agrarius* were also *B. burgdorferi* s.l.-positive.

**Table 3:** Prevalence of *B. miyamotoi* and *B. burgdorferi* s.l. in engorged ticks from rodents in Southern Hungary

Tick species and stage	*B. miyamotoi*	*B. burgdorferi* s.l.
	*(+/tested/prevalence)*	
*I. ricinus* larva pools containing 36 larvae	2/14/5.6 %^a^	4/14/11.1 %^a^
*I. ricinus* nymphs	0/5/-	1/5/20 %
*I. acuminatus* larva pools containing 52 larvae	0/13/-	4/13/7.7 %^a^
*I. acuminatus* nymph	0/1/-	1/1/100 %
*I. acuminatus* females	0/3/-	0/3/-
*D. marginatus* larva pools containing 61 larvae	0/19/-	3/19/4.9 %^a^
*D. marginatus* nymphs	0/5/-	0/5/-
*H. concinna* larva pools containing 15 larvae	0/7/-	0/7/-
*H. concinna* nymphs	0/3/-	0/3/-

^a^minimum infection prevalence

**Table 4:** Sequenced *B. miyamotoi* and *B. burgdorferi* s.l. samples from Southern Hungary

*Borrelia* species	Source	Host infection status	GenBank accession number
*B. lusitaniae*	questing *I. ricinus* nymph	-	KM657411
*B. afzelii*	*A. flavicollis* male skin	-	KM657412
*B. afzelii*	*A. agrarius* male skin	-	KM657417
*B. afzelii*	questing *I. ricinus* nymph	-	KM657413
*B. afzelii*	questing *I. ricinus* nymph	-	KM657418
*B. afzelii*	questing *I. ricinus* female	-	KM657421
*B. afzelii*	questing *I. ricinus* female	-	KM657423
*B. afzelii*	questing *I. ricinus* male	-	KM657414
*B. afzelii*	questing *I. ricinus* male	-	KM657415
*B. afzelii*	engorged *I. ricinus* larva from *A. flavicollis* female	unknown	KM657425
*B. afzelii*	engorged *I. ricinus* pool (4 larvae) from *A. flavicollis* female	uninfected	KM657426
*B. afzelii*	engorged *I. ricinus* pool (8 larvae) from *A. flavicollis* male^a^	unknown	KM657416
*B. afzelii*	engorged *I. ricinus* nymph from *A. flavicollis* male	unknown	KM657424
*B. afzelii*	engorged *I. acuminatus* pool (6 larvae) from *A. flavicollis* male^b^	unknown	KM657427
*B. afzelii*	engorged *I. acuminatus* pool (10 larvae) from *A. flavicollis* male^b^	unknown	KM657428
*B. afzelii*	engorged *I. acuminatus* nymph from *A. flavicollis* male^c^	uninfected	KM657419
*B. afzelii*	engorged *D. marginatus* pool (4 larvae) from *A. agrarius* male	uninfected	KM657422
*B. afzelii*	engorged *D. marginatus* pool (8 larvae) from *A. flavicollis* male^c^	uninfected	KM657420
*B. miyamotoi*	questing *I. ricinus* nymph	-	LC006119.1
*B. miyamotoi*	engorged *I. ricinus* pool (8 larvae) from *A. flavicollis* male^a^	unknown	LC006120.1
*B. miyamotoi*	*A. flavicollis* female spleen	-	LC006118.1

^a^co-infection

^b^from the same rodent individual

^c^from the same rodent individual

Sequencing was successful for 18 *B. burgdorferi* s.l.-positive samples: one *B. lusitaniae* was found in a questing *I. ricinus* nymph and altogether 17 *B. afzelii* were identified in questing *I. ricinus* nymphs and adults, in engorged *I. ricinus* larvae and a nymph, engorged *I. acuminatus* larvae and a nymph, and in rodent skin samples. Two *Dermacentor marginatus* engorged larva pools originating from uninfected hosts were also infected with *B. afzelii* (Table 4). We sequenced *B. miyamotoi* amplicons from one questing *I. ricinus* nymph, one engorged *I. ricinus* larva pool and a skin sample of an *A. flavicollis*.

## Discussion

Small mammals are one of the most important sources of blood meal for the subadult stages of ticks. Rodents have high metabolic and reproduction rate with relatively large body surface compared to their body weight and these small mammals are in high densities in the natural habitats. All these features make rodents suitable hosts for ticks and also suitable reservoirs for many pathogens [2]. The present study identified *B. miyamotoi* and *B. burgdorferi* s.l. from samples of different years (2011–2012) indicating that these pathogens seem to have a stable cycle in this area even surviving rodents that usually live for less than a year.

*Borrelia miyamotoi* spirochetes have been previously detected only in wild *A. argenteus* in Asia*, P. leucopus* in North-America and *My. glareolus* in Europe [16, 31, 32]. The reservoir role of *A. flavicollis* and *My. glareolus* was proven under xenodiagnostic laboratory conditions [33]. Our study provides the first evidence for the presence of *B. miyamotoi* infection in a wild *A. flavicollis* population. Further eco-epidemiological studies in other natural habitats will shed more light on the importance of one of the most common rodents in Europe, *A. flavicollis*, in the cycle of *B. miyamotoi*.

Relapsing fever spirochetes’ DNA was detectable in five samples (including *A. flavicollis* skin and spleen, questing and engorged *I. ricinus*) with a sensitive qPCR method [26]. *Borrelia miyamotoi* DNA sequencing was successful from only three samples: one questing *I. ricinus* nymph, one pooled sample containing eight *I. ricinus* larvae from an *A. flavicollis* male and one spleen removed from an *A. flavicollis* female. All three sequences were 100 % identical suggesting the circulation of the same *B. miyamotoi* genotype between natural populations of the yellow-necked field mouse and *I. ricinus*. In the case of an *A. flavicollis* male skin and one questing *I. ricinus* nymph sample the conventional PCR and sequencing were not successful, probably due to low DNA concentration. We found, altogether, 48 *B. burgdorferi* s.l. positive samples in all types of samples and from 18 of them we could also sequence the LB spirochete. Compared to Egyed et al. (2012) [39], who found 2.5 % average minimum infection prevalence of *B. burgdorferi* s.l. in questing *I. ricinus* in different collection sites in Hungary, our study, although within a much smaller sample size, shows a much higher prevalence (23.5 %). To get a better estimate of the pathogen prevalence in the area, the study has to be performed with at least ten times larger questing tick sample size. In one questing *I. ricinus* nymph we found *Borrelia lusitaniae* infection. This nymph may have fed as larva on lizards that are common hosts for *I. ricinus* larvae and potential reservoirs of these spirochetes [40, 41]. *Lacerta viridis* (green lizard)*, Lacerta agilis* (sand lizard) and *Podarcis muralis* (common wall lizard) live in this region [42] and *L. agilis* was observed also in the vicinity of our trapping sites (Balázs Velekei, personal communication). The presence of *B. lusitaniae* is of public health relevance, since this spirochete can also infect humans [43].

*Borrelia afzelii* was the most prevalent among the sequenced LB spirochetes (17/18) in the present study. This is the most widespread *Borrelia* species in Europe [27], usually maintained by rodents [33, 44]. This spirochete is probably the most important LB causative agent in Hungary [45]. In a seroepidemiological study in the neighbouring Austria a nearly linear increase of LB seroprevalence with duration of hunting activity was shown among hunters [8]. Lakos et al. [7] reported that erythema migrans occurred ten times more frequently among forestry workers than in the average population, but the rate of seropositivity was much higher (indicating frequent asymptomatic seropositivity). The hunters’ elevated risk of tick bites is obvious [11] and infections with other tick-borne pathogens, such as *Anaplasma phagocytophilum* in the neighbouring Slovakia [12], *Rickettsia* spp. in Germany [13] and tick-borne encephalitis virus in Italy [9], were also observed in this group. Thus, the presence of at least two pathogenic LB spirochetes in the Gemenc area can pose a risk of LB infection to the occupationally exposed persons.

**Fig. 1:**
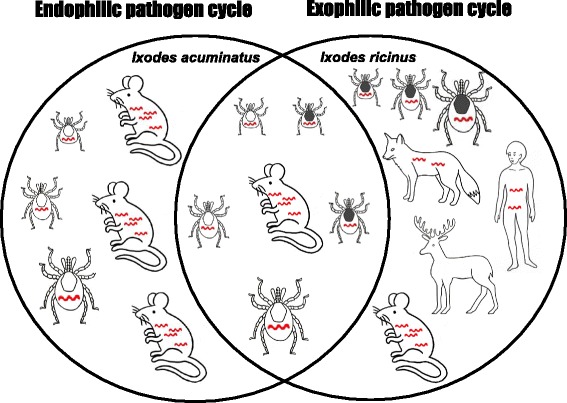
The two transmission cycles involved in the natural maintenance of *Borrelia afzelii.* Scutum of larvae, nymphs and adults of the exophilic tick, *I. ricinus* are marked with dark grey and with white colour in case of the endophilic tick, *I. acuminatus*. Red spirochetes indicate ticks and hosts that can potentially be infected with *B. afzelii*. Cervids are important tick maintenance hosts, however they are not reservoirs of LB spirochetes, thus they are known to be dilution hosts [54, 55]. Original drawings were made by Gábor Majoros

*Ixodes acuminatus* individuals are endophilic (or nidicolous) ticks. All stages of this species live in rodents’ nests, thus, being capable of maintaining a local cycle of pathogens similar to the natural cycle of *A. phagocytophilum* and *Babesia microti* with the endophilic *I. trianguliceps* [46, 47]. We found *B. burgdorferi* s.l. in one nymph and four larval pools (4/52, minimum infection prevalence: 7.7 %) of *I. acuminatus*. Rigó et al. [44] detected *B. afzelii* in an adult *I. acuminatus* female and the three sequenced *I. acuminatus* samples in the present study identified the same LB spirochete*.* Although this tick species is not currently considered a competent vector [48], our previous [44] and current data refer to an important role of *I. acuminatus* in the endophilic pathogen cycle of *B. afzelii* (Fig. 1). *Ixodes ricinus* ticks are the connecting link (bridge vectors) between the rodent’s local (nest) infection and the “world outside the nest” i.e. other vertebrate hosts including humans. Between these two *Ixodes* species we could not find any significant difference in *B. burgdorferi* s.l. prevalence. Thus, *I. acuminatus* may have similarly important role in the endophilic pathogen cycle as *I. ricinus* has in the exophilic pathogen cycle involving human infection. This double natural cycle has also been observed in the case of *B. burgdorferi* s.l. and *I. ricinus* vs. *I. hexagonus* [49] and might be a general trait for several tick-borne pathogens. Being present in two different (endophilic and exophilic) transmission cycles is clearly an evolutionarily stable strategy increasing survival of the LB spirochetes. Both of these cycles have to be considered and monitored in order to forecast and prevent human infection risk. Furthermore, *I. acuminatus* occasionally can bite humans [50] posing a direct infection threat as well.

We also found *B. burgdorferi* s.l. infection in three engorged *D. marginatus* (two larva pools and one single larva sample) that were removed from uninfected hosts. The two pools contained four and eight specimens respectively, and the bacterium identified in these samples was *B. afzelii*. One of these pools was collected from one *Borrelia*-negative *A. flavicollis* male, the other pool was removed from a *Borrelia*-negative *A. agrarius* male. The single engorged *B. burgdorferi* s.l.-positive *D. marginatus* larva was removed from an *A. flavicollis* female which was also shown to be negative for *B. burgdorferi* s.l. In previous studies, questing adult *D. reticulatus* ticks in Germany had 11.3 % prevalence of *Borrelia* spp. (detected with indirect immunofluorescence assay) [51] but in controlled experiments, American *Dermacentor* spp. were unable to transmit LB spirochete [52]. While being capable of biting humans [53], the possibility exists that *D. marginatus* is able to maintain LB spirochetes, we do not claim that *Dermacentor* spp. have an epidemiological role in LB transmission, however these findings suggest further investigation. One engorged *I. ricinus* larva pool from an unidentified rodent (n = 8 larvae) had co-infection with *B. afzelii* and *B. miyamotoi*. Cosson et al. [31] found *B. miyamotoi* co-infection with another LB spirochete, *B. garinii* in France. This indicates that *I. ricinus* might spread both pathogens even synchronously and act like a bridge vector between the most important rodent species and humans. This tick species is the key risk factor for humans acquiring most tick-borne pathogens in Europe [5], especially in areas with frequent human presence as the popular hunting ground and touristic destination in our study site.

Up until now, no human cases of *B. miyamotoi* have been reported in Hungary. The presence of this newly described pathogen in ticks and rodents, as shown here, a recent case of human infection [26], and the high seropositivity of forestry workers in the Netherlands [6] suggest, however, that human cases might occur, but they might be overlooked. The symptoms of tick-borne relapsing fever could easily be confused with those caused by other better known pathogens or with those of Lyme borreliosis.

## Conclusions

The presence of the newly described human pathogen, *Borrelia miyamotoi* in a natural habitat with frequent human visitors has important public health implications. This study is the first report of this bacterium in wild *A. flavicollis* as well as in Hungary. *Apodemus flavicollis, A. agrarius* and *My. glareolus* were found to be involved in the natural cycle of LB spirochetes. Our results suggest an important role of *I. acuminatus* ticks in the endophilic pathogen cycle of *B. afzelii*, similar to the role of *I. ricinus* in the exophilic pathogen cycle.

Forestry workers, hunters, woodcutters, gamekeepers and hikers are especially exposed to ticks in areas of intense transmission of bacteria within enzootic cycles (i.e. forests). Consequently, they have to be considered as a high risk population for both LB spirochetes and *B. miyamotoi*. In these high exposure groups, surveillance and prevention are the most crucial pillars of the protection against tick-borne pathogens like *B. burgdorferi* s.l. and *B. miyamotoi*.
